# Using the Immunization Information System to Determine Vaccination Coverage Rates among Children Aged 1–7 Years: A Report from Zhejiang Province, China

**DOI:** 10.3390/ijerph110302713

**Published:** 2014-03-05

**Authors:** Qian Li, Yu Hu, Yanpeng Zhong, Yaping Chen, Xuewen Tang, Jing Guo, Lingzhi Shen

**Affiliations:** 1 Institute of Immunization and Prevention, Zhejiang Center for Disease Control and Prevention, Hangzhou 310051, China; E-Mails: qianli@cdc.zj.cn (Q.L.); ypchen@cdc.zj.cn (Y.Z.); xwtang@cdc.zj.cn (X.T.); jguo@cdc.zj.cn (J.G.); lzhshen@cdc.zj.cn (L.S.); 2 Institute of Technology Development, Suzhou Shensu Automatic Technology CO, LTD., Shenyang 110000, China; E-Mail: zyp0269@163.com

**Keywords:** national immunization program, immunization information system, coverage, birth cohort, migrant, timeliness

## Abstract

*Background:* The Zhejiang Immunization Information System (ZJIIS) was established in 2004. This study described the coverage rates of NIP vaccines in Zhejiang Province using the ZJIIS. *Methods:* Children aged 1–7 years (born from 1 January 2005 to 31 December 2011) registered in ZJIIS were enrolled in this study. All immunization records were obtained from the ZJIIS on 31 December 2012. The cohort method had been used for identifying trends and patterns in vaccine administration. Immunization coverage estimates were analyzed for both individual NIP vaccines and “Fully immunized” by age group, birth cohort, immigration status, and geography area. We also examined the timeliness vaccination for the 2010 birth cohort. Results: A total of 3,579,896 children were registered in ZJIIS. All the vaccines and doses which scheduled to be given at ≤12 months of age exceeded 90%. There was substantial decrease trend in the vaccines scheduled at >12 months of age and most of these vaccines were below 90%. The coverage of migrant children was lower than for resident children and the coverage of WenZhou (WZ), Zhoushan (ZS) and TaiZhou (TZ) was lower than other municipalities for most of vaccines across all the birth cohorts. Nearly 20%–30% of children of 2010 birth cohort delayed for the primary series vaccination scheduled at ≤12 months of age, especially among migrant children. *Conclusions:* The ZJIIS is useful in tracking vaccine coverage of children aged 1–7 years and the data provided by ZJIIS reflected the fact that NIP delivery was improving in Zhejiang Province, while identifying some areas for improvement. We recommend continuing surveillance to estimate of vaccine coverage through ZJIIS. Immunization strategies such as Assessment, Feedback, Incentives, and Exchange program, reminder/recall activity, home visits, school entry requirements and school-based clinics could be used to reach a higher coverage of the population.

## 1. Introduction

Immunization is one of the most successful tools available for the prevention of infectious diseases. Through the utilization of vaccines, the public health achievements of the 20th century have included eradication of smallpox and substantial decrease in the incidence and mortality of vaccine-preventable diseases such as hepatitis B, polio and measles [1]. Many countries have established National Immunization Programs (NIPs) in order to produce public health leadership in vaccination practices and to provide technical and financial support for vaccination recommendations.

The Chinese NIP was started since 1978. With a long-standing history of more than 30 years, the Chinese NIP has provided efforts to accommodate the needs of children at risk of vaccine-preventable diseases. The Chinese NIP stipulates that children aged <7 years should be vaccinated with the following vaccines (Table 1) since 2008 [2]: a birth dose of Bacille Calmette-Guérin (BCG), four doses of oral Poliovirus live attenuated Vaccine (PV), four doses of Diphtheria-Pertussis-Tetanus Vaccine (DPT), three doses of Hepatitis B Vaccine (HepB), two dose of Measles Containing Vaccine (MCV), two doses of Japanese Encephalitis live attenuated Vaccine (JEV), one dose of Hepatitis A live attenuated Vaccine (HepA), two dose of Meningococcal Polysaccharide Vaccine-type A (MPV-A), one dose of MPV-type A and C (MPV-AC), and one dose of Diphtheria-Tetanus Vaccine (DT).

The NIP coverage was measured using the ICS, always conducted by the National Center for Disease Control and Prevention (CDC), and the multi-stage Probability Proportionate to Size (PPS) sampling method was usually adopted [3]. The ICS data are useful in determining overall trends of children’s vaccine uptake, but there are still limitations. It takes a long time to collect the target sample size per area and, as a result, ICS data are reported for the previous year, leaving a gap between changes in the vaccination schedule and evaluation. Although ICS coverage estimates are precise at the national level, estimates for province and local areas should be interpreted cautiously because of smaller sample sizes and wider confidence intervals. Further, national-level ICS coverage estimates are not routinely performed.

Without detailed, real-time data, a NIP’s ability to implement new strategic initiatives to increase coverage is limited. Monitoring the coverage rate of new vaccines is critical to understanding where future resources and efforts need to be directed [4]. Ideally, Immunization Information Systems (IIS) with cyber technology, which are used to collect immunization information for each child, such as the Zhejiang Immunization Information System (ZJIIS), provide a supplementary data by providing province or sub- province level data and a timely surveillance system that allows NIP to analyze the impact of strategies, such as school immunization requirements or supplementary immunization activities. In this study, we analyzed information from the ZJIIS to determine the number, coverage rates and trends of each vaccine administered, including rates by age group or geographical areas and compare the coverage rate among migrant children and resident children.

**Table 1:** Recommended children aged <7 years immunization schedule in China.

Vaccine	Age												
	Birth	1 m ^a^	2 m	3 m	4 m	5 m	6 m	8 m	18 m	24m	3 y ^b^	4 y	6 y
BCG	Dose1												
HepB	Dose1	Dose2					Dose3						
PV			Dose1	Dose2	Dose3							Dose4	
DPT				Dose1	Dose2	Dose3			Dose4				
MCV *								Dose1	Dose2				
HepA									Dose1				
JEV								Dose1		Dose2			
MPV-A **^#^**							Dose1 and Dose2						
MPV-AC											Dose1		Dose2
DT													Dose1

Notes: **^a^** m = month; **^b^** y = year; * the 1st dose of MCV use Measles-rubella combined live attenuated vaccine and the 2nd dose of MCV use Measles-mumps combined live attenuated vaccine; **^#^** 2 doses of MPV-A are scheduled from 6–18 months of age with an interval ≥3 months.

## 2. Methods

### 2.1. Zhejiang Immunization Information System

The ZJIIS is a computerized information system maintaining immunization data for children aged <7 years living in Zhejiang Province. All the immunization clinics were enrolled in the ZJIIS since 2004. ZJIIS contains a client application software deployed in every immunization clinics and a web-based management platform deployed in Zhejiang provincial CDC. The initial objectives of the ZJIIS were to enable the provincial, municipal, county level health bureaus and CDCs to better manage immunization programs and to increase coverage rates, to interrupt disease transmission, to enable vaccination providers rapidly detect local and provincial changes in vaccination coverage in real time, to develop recall-reminder systems, and to provide coverage data at regular intervals by age, vaccine and region, for program management and targeted immunization efforts.

All the children, including migrant children, are registered in ZJIIS at their first point of contact with immunization clinics and have been given a unique identification number in the ZJIIS. According to the vaccination information management regulation of Zhejiang Province issued by the Health Bureau of Zhejiang Province in 2004, the immunization physician should enter the child’s demographic information and historical immunization information into the client application software of ZJIIS, and the update immunization information(or any changes to the child’s demographic information) would also be entered after an immunization event within one month. All those information on children would be directly sent to the ZJIIS through the Internet in real time. Due to high provider participation and timely data, the ZJIIS data provide in-depth, real-time answers about the uptake of NIP vaccines that help programmatic decision-making when used in conjunction with the CIS.

### 2.2. Measuring Immunization Coverage

Children aged 1–7 years (born from 1 January 2005 to 31 December 2011) registered in ZJIIS were enrolled in this study. All immunization records of target children were queried from the provincial database of ZJIIS [5] on 31 December 2012. The cohort method had been used for calculating coverage at population or geography level [6]. Cohort immunization status was assessed at 12 months of age (for vaccines due at 8 months), 24 months of age (for vaccines due at 18 months), 5 years of age (for vaccines due at 4 years) and 7 years of age (for vaccines due at 6 years). If a child’s records indicated receipt of the last dose of a vaccine that requires more than one dose to complete the series, it was assumed that earlier vaccinations in the sequence have been given. This assumption has been shown to be valid.

Three-month birth cohorts were used for time trend analysis, while 12-month wide cohorts were used for other analysis such as immigration status or timeliness coverage analysis. These cohorts are children born between January 1, and December 31, 2011 for the 2011 birth cohort; children born between 1 January and 31 December 2010 for the 2010 birth cohort; children born between 1 January and 31 December 2007 for the 2007 birth cohort; children born between 1 January and 31 December 2005 for the 2005 birth cohort.

Immunization coverage estimates were calculated for both individual NIP vaccines and “Fully immunized” according to the specific age of children [7]. The proportion of children designated as “fully immunized” was calculated using the number of children completely immunized with the vaccines of interest by the designated age as the numerator, and the total number of ZJIIS-registered children in the age cohort as the denominator. “Fully immunized” at 12 months of age (1:3:3:3:1:1 series) was defined as a child having a record on ZJIIS of 1 dose of BCG, 3 doses of Hep B, 3 doses of PV, 3 doses of DPT, 1 dose of MCV and 1 dose of JEV. “Fully immunized” at 24 months of age (1:3:3:4:2:1:1:2 series) was defined as a child having a record on ZJIIS of 1 dose of BCG, 3 doses of Hep B, 3 doses of PV, 4 doses of DPT, 2 doses of MCV, 1 dose of JEV, 1 dose of Hep A, and 2 doses of MPV-A. “Fully immunized” at 5 years of age (1:3:4:4:2:2:1:2:1 series) was defined as a child having a record on ZJIIS of 1 dose of BCG, 3 doses of Hep B, 4 doses of PV, 4 doses of DPT, 2 doses of MCV, 2 doses of JEV, 1 dose of Hep A, 2 doses of MPV-A and 1 dose of MPV-AC. “Fully immunized” at 7 years of age (1:3:4:4:2:2:1:2:2:1 series) was defined as a child having a record on ZJIIS of 1 dose of BCG, 3 doses of Hep B, 4 doses of PV, 4 doses of DPT, 2 doses of MCV, 2 dose of JEV, 1 dose of Hep A, 2 doses of MPV-A, 2 doses of MPV-AC and 1 dose of DT.

### 2.3. Timeliness

Timely immunization was defined as receipt of a scheduled vaccine dose within 30 days of the recommended age (the 1st dose of Hep B should be vaccinated in 24 h after birth) [8]. For example, a child who received the 1st dose of PV (due at 2 months or 60 days of age) when he or she was more than 90 days of age was classified as not timely immunized (*i.e.*, late for the dose). For descriptive purposes, we categorized the outcome measure for each dose as either vaccine dose “no delay” (timely immunized), “delay of between 1 to 3 months”, or “delay greater than 3 months”. Doses received “too early” (greater than 1 month or 30 days prior to when it was due), and doses never administered or recorded were excluded. Children included in the timeliness analysis were assessed at least one year after doses were due, to allow time for late vaccinations to be recorded [9]. Timeliness has been examined for the 2010 birth cohort and for vaccines requiring both multiple doses (PV, DPT, Hep B) and a single dose (MCV) under 12 months of age in this study. The interval between doses was not evaluated. Timeliness of different vaccines and doses was also compared by plotting the cumulative percentage receiving each vaccine dose by age, with the proportion immunized set as 100%.

### 2.4. Immigration Status

Immigration status on the ZJIIS is recorded as “migrant child from other province”, “migrant child from other municipalities of Zhejiang Province”, or “resident child”, as reported by the immunization clinics to the ZJIIS. For this study we considered two categories of children: “resident child” and “migrant child”, “migrant child from other province” and “migrant child from other county in Zhejiang province” were presumed to be “migrant child”. Migrant child from abroad were not included in this study.

### 2.5. Statistical Analysis

For data analysis, we organized the database as an Excel (Microsoft Office Excel 2010) file. Coverage rates were calculated by use of the Excel program.

### 2.6. Ethical Considerations

This study was approved by the Ethical Review Board of Zhejiang Provincial Center for Disease Control and Prevention. All the data were anonymous when we exported them from ZJIIS and kept confidential without individual identifiers.

## 3. Results

### 3.1. Over All Vaccine Coverage

During the study period, a total of 3,579,896 children were registered in ZJIIS and 40.7% (1,457,299) of these children were migrant. Coverage rates for individual vaccines and “fully immunized” in 2012 for children aged 1–7 years at the four milestone ages of 12 months, 24 months, 5years and 7 years were summarized in Table 2, Table 3, Table 4 and Table 5. All the vaccines and doses which scheduled to be given at ≤12 months of age exceeded the NIP target of 90% in both the provincial level and municipal level. There was substantial decrease trend in the vaccines and doses scheduled to be given at >12 months of age and all those vaccines and doses were below the NIP target.

**Table 2:** Percentage of children immunized at 12 months of age *, by vaccine and territory.

Vaccine	Coverage by Municipality ^# ^(%)										
	HZ	NB	WZ	JX	SX	JH	QZ	ZS	TZ	LS	Total
No. of children	543,710	579,937	707,463	241,947	245,362	429,378	131,836	48,392	521,945	129,926	3,579,896
BCG	99.5	99.5	98.0	99.5	99.4	98.6	98.7	99.6	97.9	99.1	98.8
Hep B ≥ 3	99.7	99.5	96.6	99.6	99.4	98.2	97.9	99.3	95.3	98.5	98.1
PV ≥ 3	99.7	99.7	97.6	99.8	99.7	98.2	98.0	99.3	95.6	99.0	98.1
DPT ≥ 3	99.6	99.4	96.6	99.5	99.5	99.4	98.0	99.2	95.1	98.7	98.4
MCV ≥ 1	99.8	99.6	97.1	99.8	99.6	98.8	98.3	99.6	96.4	98.8	98.6
JEV ≥ 1	99.2	99.1	91.8	99.3	98.6	97.1	96.0	97.1	90.8	96.8	96.0
Fully immunized ^▲^	98.7	98.5	90.5	98.7	97.9	96.0	95.3	96.7	97.9	95.8	95.1

Notes: * Target children born from1 January 2005 to 31 December 2011; **^#^** HZ: HangZhou, NB: NingBo, WZ: WenZhou, JX: JiaXing, SX: ShaoXing, JH: JinHua, QZ: QuZhou, ZS: ZhouShan, TZ: TaiZhou, LS: LiShui; ^▲^ 1:3:3:3:1:1 series.

**Table 3:** Percentage of children immunized at 24 months of age *, by vaccine and territory.

Vaccine	Coverage by Municipality ^# ^(%)										
	HZ	NB	WZ	JX	SX	JH	QZ	ZS	TZ	LS	Total
No. of children	442,547	482,087	588,192	196,450	204,151	350,668	112,304	40,261	439,350	110,494	2,966,504
BCG	99.5	99.4	97.8	99.6	99.4	98.5	98.6	99.6	97.7	99.1	98.7
Hep B ≥ 3	99.6	99.4	96.4	99.6	99.4	98.1	97.8	99.2	94.9	98.5	98.0
PV ≥ 3	99.6	99.6	97.4	99.8	99.6	98.2	97.9	99.2	95.3	99.0	98.0
DPT ≥ 4	98.1	97.5	85.1	98.6	97.4	96.0	95.1	94.9	87.6	93.8	95.1
MCV ≥ 2	99.0	99.1	91.6	99.4	98.1	74.7	76.5	76.4	94.4	95.3	93.2
JEV ≥ 1	99.2	99.1	91.4	99.3	98.5	97.1	95.7	96.8	90.1	96.7	95.8
Hep A	96.9	97.9	75.8	97.1	82.1	80.5	81.0	80.2	73.7	87.1	79.7
MPV-A ≥ 2	85.5	79.7	75.7	86.9	82.7	81.5	78.6	79.0	65.5	80.0	77.1
Fully immunized ^▲^	84.0	78.5	64.8	85.1	70.5	61.6	67.2	62.1	64.9	73.4	73.6

Notes: * Target children born from 1 January 2005 to 31 December 2010; **^#^** HZ: HangZhou, NB: NingBo, WZ: WenZhou, JX: JiaXing, SX: ShaoXing, JH: JinHua, QZ: QuZhou, ZS: ZhouShan, TZ: TaiZhou, LS: LiShui; ^▲^ 1:3:3:4:2:1:1:2 series.

**Table 4:** Percentage of children immunized at 5 years of age *, by vaccine and territory.

Vaccine	Coverage by Municipality ^# ^(%)										
	HZ	NB	WZ	JX	SX	JH	QZ	ZS	TZ	LS	Total
No. of children	194,272	223,844	277,134	83,544	101,672	161,333	58,487	18,029	220,077	56,922	1,395,314
BCG	99.3	99.2	97.1	99.5	99.3	98.4	97.9	99.5	96.9	99.1	98.3
Hep B ≥ 3	99.4	99.2	94.5	99.6	99.2	97.9	96.8	99.0	93.4	98.1	97.1
PV ≥ 4	94.0	97.1	68.1	98.3	92.5	94.2	91.1	94.3	81.1	75.8	86.6
DPT ≥ 4	97.5	96.1	79.9	98.5	96.1	93.1	92.7	93.4	83.4	92.2	90.5
MCV ≥ 2	98.4	98.4	86.8	99.3	96.6	76.3	83.2	79.1	67.3	93.1	87.5
JEV ≥ 2	93.0	96.9	68.3	96.6	93.1	87.9	83.2	87.3	70.9	82.1	83.9
Hep A	95.4	96.8	65.6	95.3	73.0	63.8	66.5	61.9	57.0	81.4	76.1
MPV-A ≥ 2	85.5	62.5	60.1	72.3	67.6	86.3	79.2	81.0	66.5	66.9	70.7
MPV-AC ≥ 1	94.7	91.6	62.4	97.5	90.7	68.9	83.0	82.2	88.0	74.7	82.2
Fully immunized ^▲^	63.1	58.8	36.5	68.2	49.1	47.1	52.7	49.5	38.6	47.0	49.4

Notes: * Target children born from 1January 2005 to 31 December 2007. **^#^** HZ: HangZhou, NB: NingBo, WZ: WenZhou, JX: JiaXing, SX: ShaoXing, JH: JinHua, QZ: QuZhou, ZS: ZhouShan, TZ: TaiZhou, LS: LiShui; ^▲^ 1:3:4:4:2:2:1:2:1 series.

**Table 5:** Percentage of children immunized at 7 years of age *, by vaccine and territory.

Vaccine	Coverage by Municipality ^# ^(%)										
	HZ	NB	WZ	JX	SX	JH	QZ	ZS	TZ	LS	Total
No. of children	59,045	69,663	87,199	24,468	35,191	51,013	20,432	5,254	73,608	17,650	443,523
BCG	99.1	99.1	97.9	99.4	99.1	98.5	96.8	99.5	96.2	98.9	98.2
Hep B ≥ 3	99.2	99.1	93.9	99.5	98.8	98.0	95.7	99.1	93.0	97.9	96.7
PV ≥ 4	91.5	97.4	63.4	98.0	88.7	92.7	87.5	92.9	75.9	68.2	83.5
DPT ≥ 4	95.9	94.8	73.5	97.8	93.2	86.6	88.2	88.4	76.6	89.8	86.3
MCV ≥ 2	97.2	98.3	80.9	99.0	93.1	67.1	75.7	79.0	59.8	89.3	82.8
JEV ≥ 2	88.1	96.7	59.6	96.5	88.9	81.0	76.0	80.5	60.7	74.8	77.8
Hep A	93.3	96.1	59.3	92.7	68.0	19.8	28.2	17.0	20.2	76.2	59.8
MPV-A ≥ 2	23.3	26.2	34.7	25.0	21.4	82.2	67.3	70.1	55.5	41.2	41.4
MPV-AC ≥ 2	71.8	72.9	18.6	60.8	39.8	48.1	66.3	41.5	40.5	24.6	48.0
DT	78.8	91.5	30.4	96.0	79.7	77.2	75.3	23.9	22.4	48.1	60.7
Fully immunized ^▲^	15.9	20.6	9.0	16.2	8.3	9.5	17.9	9.8	6.7	11.5	12.3

Notes: * Target children born from 1 January 2005 to 31 December 2005; **^#^** HZ: HangZhou, NB: NingBo, WZ: WenZhou, JX: JiaXing, SX: ShaoXing, JH: JinHua, QZ: QuZhou, ZS: ZhouShan, TZ: TaiZhou, LS: LiShui; ^▲^ 1:3:4:4:2:2:1:2:2:1 series.

### 3.2. Coverage Trends among Birth Cohorts

**Figure 1:**
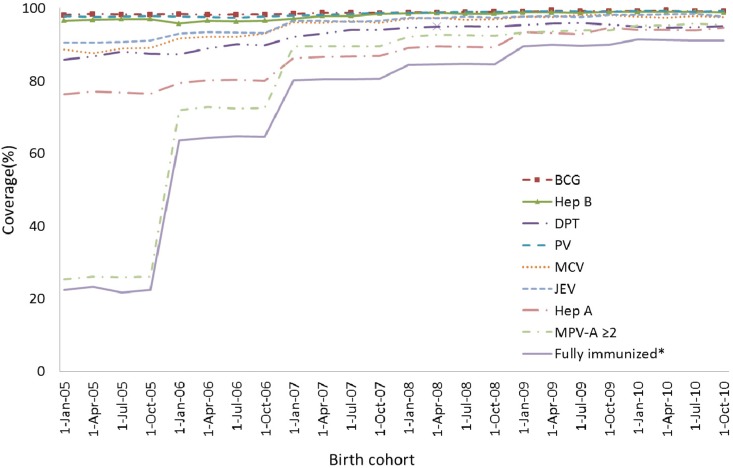
Trends in vaccination coverage estimates for individual vaccines at 12 months of age (≥3 dose of Hep B, PV and DPT, ≥1 dose of BCG, MCV, JEV, “Fully immunized”), by 3-month birth cohorts born between 1 January 2005 to 31 December 2011. Coverage assessment date was 12 months after the last birth date of each cohort.

Note: * 1:3:3:3:1:1 series.

Across all birth cohorts (2005–2011), the coverage for series vaccination such as BCG (≥1 dose), Hep B (≥3 doses), DPT (≥3 doses), PV (≥3 doses), and MCV (≥1 dose) remained over 95% and shown in Figure 1. The coverage rates for JEV (≥1 dose) and “fully immunized” at 12 months of age (1:3:3:3:1:1 series) was lower than other vaccines mentioned above, the coverage tended to increase gradually over the years. The coverage for the 1^st^ dose of JEV showed an increase from 90.7% in the 2005 birth cohort to 97.1% in the 2011 birth cohort. Although the coverage for “Fully immunized” at 12 months of age (1:3:3:3:1:1 series) in the 2005 birth cohort was low at 89.5%, the steady increase has reached coverage of 96.4% in the 2011 birth cohort.

**Figure 2:**
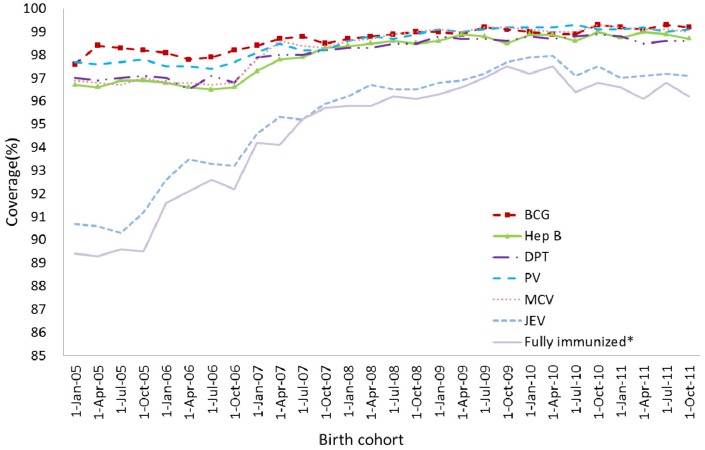
Trends in vaccination coverage estimates for individual vaccines at 24 months of age (≥4 doses of DPT, ≥3 dose of Hep B and PV, ≥2 dose of MCV and MPV-A, ≥1 dose of BCG, JEV, Hep A, “Fully immunized”), by 3-month birth cohorts born between 1 January 2005 to 31 December 2010. Coverage assessment date was 12 months after the last birth date of each cohort.

Note: * 1:3:3:4:2:1:1:2 series.

Figure 2 showed the time trend in series vaccination coverage for individual vaccines and “Fully immunized” at 24 months of age (1:3:3:4:2:1:1:2 series). The coverage of BCG (≥ 1 dose), Hep B (≥3 doses), PV (≥3 doses) had were significantly higher than other vaccines and remained above 95%. Coverage rates series vaccination like DPT (≥4 doses), MCV (≥2 doses), JEV (≥1 doses) and Hep A (≥1 dose) rose steadily from below 90% in the 2005 birth cohort to 98% in the 2010 birth cohort. Although coverage rates were lowest for MPV-A series (≥2 doses) and “fully immunized” in the 2005 birth cohort, there was a marked increase in the 2006 birth cohort and both of them finally reached 90% target in the 2010 birth cohort.

### 3.3. Coverage Difference between Resident Children and Migrant Children

Immunization coverage was lower for migrant children than resident children in 2005, 2007, 2010 and 2011 birth cohort for most vaccines with the difference being largest in 2005 birth cohort (shown in Table 6). The difference in coverage for vaccines scheduled at ≤12 months of age had been relatively consistent and lower than the difference of the coverage among vaccines scheduled at an elder age. The coverage differential between resident and migrant children for individual vaccines varied among four age milestones. The biggest difference of coverage between resident and migrant children was found in MPV-AC (≥2 doses) in 2005 birth cohort.

**Table 6:** Immunization coverage estimates by 12-month wide cohorts, vaccines and immigration status, 2012.

Vaccine	Birth Cohort *	No. of Children	Coverage by Immigration Status(%)	
			Resident Child	Migrant Child
BCG ≥ 1	2011	613,392	99.5	98.9
	2010	551,143	99.4	98.9
	2007	492,494	99.0	97.9
	2005	443,523	99.0	96.1
Hep B ≥ 3	2011	613,392	99.2	98.2
	2010	551,143	99.2	98.5
	2007	492,494	98.4	97.1
	2005	443,523	97.8	93.8
PV ≥ 3	2011	613,392	99.4	98.9
	2010	551,143	99.3	99.1
PV ≥ 4	2007	492,494	88.8	83.8
	2005	443,523	78.3	74.0
DPT ≥ 3	2011	613,392	99.1	98.2
DPT ≥ 4	2010	551,143	96.5	92.9
	2007	492,494	95.6	90.6
	2005	443,523	90.0	77.8
MCV ≥ 1	2011	613,392	99.3	98.7
MCV ≥ 2	2010	551,143	98.6	96.5
	2007	492,494	97.0	94.3
	2005	443,523	90.4	83.0
JEV ≥ 1	2011	613,392	98.1	96.1
	2010	551,143	98.6	97.3
JEV ≥ 2	2007	492,494	91.5	84.8
	2005	443,523	80.3	70.9
Hep A ≥ 1	2010	551,143	95.5	92.2
	2007	492,494	87.6	84.0
	2005	443,523	79.0	70.6
MPV-A ≥ 2	2010	551,143	97.3	93.9
	2007	492,494	92.9	83.5
	2005	443,523	24.2	30.8
MPV-AC ≥ 1	2007	492,494	87.8	82.7
MPV-AC ≥ 2	2005	443,523	52.6	38.5
DT ≥ 1	2005	443,523	61.9	55.3

Notes: * 2011: children born between 1 January and 31 December 2011; 2010: children born between 1 January and 31 December 2010; 2007: children born between 1 January and 31 December 2005; 7 year: children born between 1 January and 31 December 2005.

For 2011 birth cohort, the overall proportion of migrant children fully vaccinated at 12 months of age was 95.0%, compared with 97.6% for resident children (shown in Table 7). Although coverage was lower among migrant children in all municipalities, the extent of the difference varied, reaching a 5.5% point difference in JH municipality and a 5% point difference in TZ municipality. However, for 2010 and 2007 birth cohorts, the disparity between resident and migrant children had increased provincially to be 5.6% points and 11 percentage points lower for migrant children. There was dramatic variation among individual municipalities in 2007 birth cohort, ranging from 3.5% points lower in LS municipality to 35.4% points lower in ZS municipality. For 2005 birth cohort, the proportion of children recorded as “Fully immunized” was the lower than any other birth cohorts. At the provincial level, the coverage for resident and migrant children was only 13.6% and 11%, while the coverage difference among individual municipalities had been relatively stable in less than 8% points.

**Table 7:** Percentage of children “fully immunized” at 12 months, 24 months, 5 years and 7 years * of age, by 12-month wide cohorts, immigration status and municipality, 2012.

Birth Cohort ^#^	Immigration Status	“Fully Immunized” Coverage by Municipality ^▲ ^(%)										
		HZ	NB	WZ	JX	SX	JH	QZ	ZS	TZ	LS	Total
2011	Resident child	99.1	99.3	94.3	99.6	99.4	99.1	97.8	99.3	96.0	96.8	97.6
	Migrant child	98.1	98.5	90.8	97.4	97.1	93.6	95.1	94.9	91.0	94.2	95.0
2010	Resident child	98.2	98.1	84.8	99.5	98.7	97.7	94.8	97.0	90.2	91.0	93.8
	Migrant child	94.6	93.6	79.3	94.6	94.4	87.7	89.2	80.5	78.1	86.7	88.2
2007	Resident child	92.0	97.0	52.8	98.2	72.5	79.7	79.1	78.7	64.2	62.5	75.1
	Migrant child	79.5	78.0	43.9	84.7	61.5	59.5	67.9	43.3	42.8	66.0	64.1
2005	Resident child	17.0	24.4	9.3	16.5	8.6	12.0	10.2	9.7	7.2	11.5	13.6
	Migrant child	12.2	16.3	7.6	14.5	5.3	8.5	8.6	8.6	4.1	11.4	11.0

Notes: * “Fully immunized”: 1:3:3:3:1:1 series for 2011 birth cohort, 1:3:3:4:2:1:1:2 series for 2010 birth cohort, 1:3:4:4:2:2:1:2:1 series for 2007 birth cohort, 1:3:4:4:2:2:1:2:2:1 series for 2005 birth cohort; **^#^** 2011: children born between 1 January and 31 December 2011; 2010: children born between 1 January and 31 December 2010; 2007: children born between 1 January and 31 December 2005; 7 year: children born between 1 January, and 31 December 2005; ^▲^ HZ: Hangzhou, NB: Ningbo, WZ: Wenzhou, JX: Jiaxing, SX: Shaoxing, JH: Jinhua, QZ: Quzhou, ZS: Zhoushan, TZ: Taizhou, LS: Lishui.

### 3.4. Coverage Difference among Geographic Areas

Table 2, Table 3, Table 4and Table 5 described the coverage for individual vaccination and “fully immunized” by geographic areas. Overall coverage for most vaccines and “fully immunized” scheduled to be given at ≤12 months of age was relatively constant across the geographic areas. However, vaccination coverage for vaccines and “fully immunized” scheduled to be given at 12–24 months of age was lower in WZ and TZ municipalities. The same pattern was also found in the vaccines scheduled at 3years and 6years of age.

### 3.5. Timeliness of Immunization

Timeliness has been examined for the 2010 birth cohort and for primary series vaccination scheduled to be given at ≤12 months of age (except BCG and JEV). Nearly 20%–30% of children delayed for their vaccination (shown in Table 8). Timely vaccination coverage for Hep B_3_, PV_3_, DPT_3_ and MCV_1_ were lower than the timely vaccination coverage for Hep B_1_. The proportion with short delays (1–3 months) was higher than that for long delays (≥3 months). The proportion of delay ≥3 months occurred more frequent for PV_3_, while the proportion of delay for 1–3 months occurred more frequent for MCV_1_.

**Figure 3:**
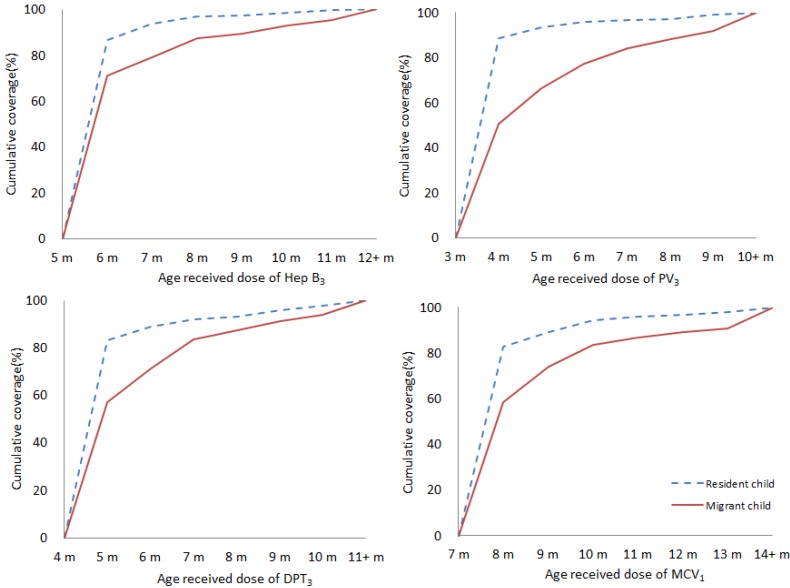
Timeliness of Hep B_3_, PV_3_, DPT_3_ and MCV_1_ by immigration status- cohort born in 2010. Percentage coverage = number of children who received vaccine dose at particular ages/the total number of children who received the vaccine dose (doses never administered or recorded were excluded.

Notes: Hep B_1_= 1st dose of Hep B, Hep B_3_= 3rd dose of Hep B, PV_3_= 3rd dose of PV, DPT_3_= 3rd dose of DPT, MCV_1_= 1st dose of MCV.

Compared with resident children, migrant children were more likely to have delayed vaccination (shown in Figure 3). For the 3rd of Hep B, there was greater delay for migrant children than resident children, with a 15.3% differential of timely vaccination at 6 months of age. The same pattern was also found in the 3rd dose of PV, 3rd dose of DPT and 1st dose of MCV. The biggest difference of timely vaccination between resident and migrant children was the 3rd dose of DPT at 3 months of age (37.9%).

**Table 8:** Vaccination delay for the 2010 birth cohort.

Vaccine	No Delay	Delay for 1–3 Months	Delay ≥ 3 Months
Hep B_1_	85.3%	N.A.	N.A.
Hep B_3_	78.5%	13.4%	8.1%
PV_3_	68.8%	17.3%	13.9%
DPT_3_	70.0%	17.7%	12.3%
MCV_1_	70.1%	18.6%	11.3%

Notes: N.A.: Not available; Hep B_1_= 1st dose of Hep B; Hep B_3_= 3rd dose of Hep B, PV_3_= 3rd dose of PV, DPT_3_= 3rd dose of DPT, MCV_1_= 1st dose of MCV.

## 4. Discussion

Since its inception, the ZJIIS has grown to hold records for over 10 million children and receives report from over 2,000 immunization service providers. The functionality of the ZJIIS has been enhanced over time in 2006, 2008 and 2010 so that it now has many features of an “ideal” immunization register, including enrollment at birth, a unique personal identifier, information on vaccine dose, date, Lot No. and provider, and mechanisms for aggregating data regionally and provincially. The main alternative to an immunization register is a periodic cross-sectional ICS. These can be conducted by telephone, such as occurs with the U.S. National Immunization Survey [10], which is of sufficient size to estimate immunization coverage rates for children aged 19 to 35 months in all 50 states in the USA, or by personal interview at the household level, such as the surveys conducted by the Zhejiang provincial CDC before. Compared with those two methods, the ZJIIS tends to under-estimate the coverage due to incomplete provider reporting, while the ICS would tend to over-estimate coverage due to inherent selective bias of missing the migrant children with lower coverage. A strength of ICS is that a wide variety of socio-demographic information about children and their caregivers can be collected [11]. The only socio-demographic information collected by the ZJIIS is the age, sex and migrant status of the child, limiting the scope of immunization coverage research which can be conducted using ZJIIS-derived data. An advantage of the ZJIIS is that it can be considered to be a census of children <7 years in Zhejiang province rather than a sample of the target population, as used in ICS methodology. Although children not captured by the ZJIIS may differ in their immunization status, it should have little impact on coverage rates as an estimated over 95% of children have already registered in the ZJIIS.

In China, although challenge in financing having limited the drive to expand the NIP vaccines, gradual progress has been made in to increase the coverage of NIP vaccines, including the financial incentives to providers with a 2–5 CNY per doses varied among different municipalities since 2009 [12], vaccination training program toward the immunization staff and improving the quality and accessibility of the vaccination service in all immunization clinics since 2009. By implementing those interventions, an increase in coverage for NIP vaccines was expected. The coverage of vaccines scheduled at ≤12 months ranged between 96%–98.8%, which was higher than previous province-wide ICS conducted in 2011 [13]. But we still found that, across all birth cohorts, coverage for booster doses (schedule at >12 months) was lower than that of primary series doses (scheduled at ≤12 months). Some children who initiated the vaccines that required more than one dose and eligible to complete the series had not received all the doses. The first reason for the low coverage could be loss of vaccination awareness as children gets older, and the second one may be the frequency of recommended well-child visits declined after 12 months of age, which was identified as a barrier to immunization in previous study [14]. There are different approaches to overcome this low coverage in older children: first, the immunization providers should review their immunization information system record at each clinic visit, and the providers should incorporate reminder/recall activity into their practice; second, the supervisor of NIP should adopt the Assessment, Feedback, Incentives and Exchange (AFIX) program help improve childhood immunization rates, which had been proved to be a cost-effective way to educate providers and increase vaccination rates in USA [15]; third, strategies such as school immunization clinics and expanding the day-care centers/elementary schools vaccination requirements could help immunization providers reach a greater coverage for vaccines scheduled to be given at an elder age.

Since 2008, the Chinese NIP included the 2nd dose of JEV, the 2nd dose of MPV-A, two doses of MPV-AC and one dose of Hep A. The decision was made base on the consensus drawn by the experts from Chinese Advisory Committee on Immunization Practice (CACIP) [12]. For the children aged ≥24 months, the ZJIIS data shown that the vaccination coverage for newly inclusion vaccines was notably lower than the traditional vaccines. The coverage for traditional vaccines ranged between 93.2% and 98.7%, whereas the coverage for newly inclusion vaccines ranged between 77.1% and 79.7%. This finding is similar to a report from the USA. [16] and Switzerland [17] that described the lower vaccination coverage for newly introduced vaccines compared to the traditionally-recommended vaccines, which may then result in the accumulation of pocket of susceptible population, and consequent increase of the risk of disease resurgence. The main reasons for this difference included two aspects: first, it was probably that the public perception and training of healthcare professionals require additional time as newly vaccines were included in the NIP; second, the utilization of these vaccines may not be adequate while the support from public health system is limited and parents’ concern on effect and safety of the newly included vaccines. This delay in widespread use of the newly included vaccines may be accelerated by implementing health education program or social-propaganda for providers and parents on the effect and safety of NIP vaccines included recently and political commitment on expanding the financial and resources support to immunization service providers.

Coverage for migrant children remained sub-optimal for most vaccines in the study period, while the difference reduced significantly in 2011 birth cohort. Lower coverage for vaccines targeted at migrant children has been a relatively consistent finding using a range of different methods [12,18,19]. So, it had a significant impact on the vaccination coverage for the whole population as over 40% of the registered children were migrant. Compared with resident children, migrant children lived in a poorer rural areas, including in a lower socio-economic developing level, their family had a lower income, and their parents’ had little knowledge of health, so few of migrant people considered it was important to have been fully immunized for their child. Besides, poor identification of migrant children by immunization providers were also likely to be an important contributing factor.

Vaccination coverage continues to vary across areas and it was lower in some municipalities such as WZ, ZS and TZ. Clusters of unvaccinated children leave communities vulnerable to outbreaks of disease. The continued occurrence of measles outbreaks among unvaccinated persons in Zhejiang province underscores the importance of maintaining uniformly high coverage to prevent transmission of vaccine preventable diseases like polio or measles. NIP financial policy as well as differences by municipality in factors such as population characteristics, scale of migrant children, immunization program activities, accessibility to immunization service, vaccination requirements for day-care centers/elementary schools might contribute to variations in vaccination coverage.

Although coverage data reveal that many children eventually complete the scheduled vaccination series by the 24-month milestone, some of which still did not do so in a timely manner. Vaccination delay in 2010 birth cohort as measured in this report for vaccines assessed at 12 and 24 months of age had improved little compared with the previous province-wide ICS conducted in 2005 [20]. The main reasons included that: first, children’s caregivers who did not have their child vaccinated were the least aware of the necessity for vaccination and its schedule [21]; second, children who were delayed or unvaccinated probably had difficulties with other preventive and primary care such as screening and well-child care visits and did not catch up their vaccination schedule even at a later age. Immunization at the earliest appropriate age should be a public health goal for areas such as Zhejiang Province where high levels of vaccine coverage at milestone ages have been achieved.

This study is subjected to two limitations. First, the ZJIIS contains more children aged 1–7 years than census data suggest are in Zhejiang Province. This discrepancy may be due to duplicate records and because children who move to Zhejiang Province and receive an immunization are added to the ZJIIS, but the ZJIIS does not currently have a way to track children who move out. Therefore, the ZJIIS overestimates population size and, as a result, underestimates immunization rates when ZJIIS population estimates are used as a denominator. Second, only children with immunization record in ZJISS were included in this study, therefore, these populations are already at greater likelihood of being vaccinated.

## 5. Conclusion

Data provided in this report by ZJIIS reflected the successful delivery of NIP in Zhejiang Province, while identifying some problems for improvement. The 2012 results should be considered a baseline against which future trends in coverage can be evaluated. We recommend continuing surveillance on the total population and subpopulations (e.g., resident/migrant and geographic) estimate of vaccine coverage through ZJIIS to assess the vulnerability to the vaccine-preventable diseases among Zhejiang Province. The coverage differences described in this report may provide guidance for NIP decisions with respect to identifying vulnerable populations and expanding the NIP to fulfill the needs of under-vaccinated populations. Parents and health-care providers should work to sustain high coverage and improve coverage for the more recently recommended vaccines and those that require booster doses scheduled at >12 months of age. Along with interventions already underway, immunization strategies such as Assessment, Feedback, Incentives, and Exchange (AFIX) program, reminder/recall activity, home visits, school entry requirements and school-based clinics could be used to reach a higher coverage of the population.
